# Indoor and Outdoor Exposure to Ultrafine, Fine and Microbiologically Derived Particulate Matter Related to Cardiovascular and Respiratory Effects in a Panel of Elderly Urban Citizens

**DOI:** 10.3390/ijerph120201667

**Published:** 2015-02-02

**Authors:** Dorina Gabriela Karottki, Michal Spilak, Marie Frederiksen, Zorana Jovanovic Andersen, Anne Mette Madsen, Matthias Ketzel, Andreas Massling, Lars Gunnarsen, Peter Møller, Steffen Loft

**Affiliations:** 1 Section of Environmental Health, Department of Public Health, Faculty of Health and Medical Sciences, University of Copenhagen, Øster Farimagsgade 5, DK-1014, Copenhagen, Denmark; E-Mails: doka@sund.ku.dk (D.G.K.); pemo@sund.ku.dk (P.M.); 2 Danish Building Research Institute, Department of Construction and Health, Aalborg University, Copenhagen, Denmark; A.C. Meyers Vaenge 15, 2450, Copenhagen, Denmark; E-Mails: mps95@drexel.edu (M.S.); mfr@sbi.aau.dk (M.F.); lbg@sbi.aau.dk (L.G.); 3 Center for Epidemiology and Screening, Department of Public Health, Faculty of Health and Medical Sciences, University of Copenhagen, Øster Farimagsgade 5, DK-1014 Copenhagen, Denmark; E-Mail: vlq961@sund.ku.dk; 4 National Research Centre for the Working Environment, Lersø Parkallé 105, 2100 Copenhagen, Denmark; E-Mail: amm@arbejdsmiljoforskning.dk; 5 Department of Environmental Science, Aarhus University, Frederiksborgvej 399, 4000 Roskilde, Denmark; E-Mails: mke@dmu.dk (M.K.); anma@dmu.dk (A.M.)

**Keywords:** ultrafine particles, indoor air pollution, ambient air pollution, PM_2.5_, bioaerosols, microvascular function, lung function, inflammation

## Abstract

To explore associations of exposure to ambient and indoor air particulate and bio-aerosol pollutants with cardiovascular and respiratory disease markers, we utilized seven repeated measurements from 48 elderly subjects participating in a 4-week home air filtration study. Microvascular function (MVF), lung function, blood leukocyte counts, monocyte adhesion molecule expression, C-reactive protein, Clara cell protein (CC16) and surfactant protein-D (SPD) were examined in relation to exposure preceding each measurement. Exposure assessment included 48-h urban background monitoring of PM_10_, PM_2.5_ and particle number concentration (PNC), weekly measurements of PM_2.5_ in living- and bedroom, 24-h measurements of indoor PNC three times, and bio-aerosol components in settled dust on a 2-week basis. Statistically significant inverse associations included: MVF with outdoor PNC; granulocyte counts with PM_2.5_; CD31 expression with dust fungi; SPD with dust endotoxin. Significant positive associations included: MVF with dust bacteria; monocyte expression of CD11 with PM_2.5_ in the bedroom and dust bacteria and endotoxin, CD31 expression with dust serine protease; serum CC16 with dust NAGase. Multiple comparisons demand cautious interpretation of results, which suggest that outdoor PNC have adverse effects on MVF, and outdoor and indoor PM_2.5_ and bio-aerosols are associated with markers of inflammation and lung cell integrity.

## 1. Introduction

The epidemiological evidence convincingly indicate that both short- and long-term exposure to ambient air pollution, especially particulate matter (PM), is associated with acute and chronic adverse health effects, including changes in lung function and asthma attacks, respiratory and cardiovascular hospital admissions, mortality and morbidity, particularly among elderly and people with previous respiratory and cardiovascular diseases [1,2,3]. PM is subdivided based on the aerodynamic diameter into PM_10_, (mass of PM with aerodynamic diameter less than 10 μm), fine (PM_2.5_, (mass of PM with aerodynamic diameter less than 2.5 µm)) and ultrafine particles (UFP, with aerodynamic diameter less than 0.1 µm). Ambient particles have different chemical composition, depending on their emission source, particle size, geographic location, meteorology, and atmospheric behavior [4,5]. Most studies have examined the association between outdoor exposure to PM_10_ or PM_2.5_ and adverse health effects, whereas there is still limited evidence about the health effects of traffic-related or indoor UFP, mainly characterized as particle number concentration (PNC) [3,6,7]. However, UFP, especially from combustion processes, have recently received more attention as possibly more harmful than larger particles, since their very small size allow them to translocate from the lungs into the circulation [8]. It has been proposed that exposure to ambient air PM may induce pulmonary and systemic inflammation, oxidative stress, altered cardiac autonomic function, altered balance between coagulation and fibrinolysis, endothelial and microvascular dysfunction, atherosclerosis progression and plaque instability [1]. Panel and cross-sectional studies have shown associations between levels of ambient air PM measured mainly as mass at monitoring stations in the preceding days and changes in prognostic markers for pulmonary and cardiovascular disease, but the results have been inconsistent regarding lung function, blood markers reflecting inflammation (C-reactive protein (CRP), leukocyte counts), cell expression of adhesion molecules and impaired endothelial function [3,9]. Assessing people’s exposure to indoor air pollution at home is important, because most people, especially those from the susceptible groups, spend 80%–90% of their day indoors, and respiratory health effects of PM is affected by the time people spend indoors and the concentration of PM in the indoor air [10,11].

The indoor UFP concentration is affected by several factors such as outdoor UFP concentration, building characteristics, infiltration and penetration factor, air exchange rates, indoor and outdoor meteorological parameters, personal behaviors, as well as indoor sources and human-related activity [12,13,14]. Indoor emission sources (cooking, candle burning, heating devices, environmental tobacco smoke, office equipment, biological sources, and human activity) can substantially contribute to the total UFP exposure in the homes [15,16,17,18,19]. Bio-aerosols are airborne particles, which consist of aerosols containing microorganisms (bacteria, fungi, viruses) or organic compounds derived from microorganisms (endotoxins, N-acetyl-β-d-glucosaminidase (NAGase), serine protease), have different size (0.3 to 100 µm) and contribute to about 5% to 34% of indoor air pollution with concentrations affected by season and ventilation rate [20]. Exposure to bacteria, endotoxin, fungi and other components found in settled dust can cause respiratory health effects including airway inflammation, exacerbation of airway and allergic diseases, and asthma, especially in children [21,22]. Studies on adults with asthma and rhinitis have shown that home indoor environmental exposure was strongly linked to lung function, health status, and disease severity [23]. So far, studies comparing outdoor and indoor PM with respect to respiratory and cardiovascular health effects are scarce and previous cross-sectional designs are susceptible to individual confounders [24,25].

In this study we took advantage of seven repeated measurements of cardiovascular and respiratory effect markers collected over 4 weeks in each of 48 elderly subjects participating in a home air filtration study to investigate associations with PM-related exposure indoors and outdoors [26]. We investigated microvascular function (MVF), lung function, blood leukocyte counts, monocyte expression of adhesion molecules, CRP as well as biomarkers of lung permeability in terms of Clara cell protein (CC16) and surfactant protein-D (SPD). Measurement of endothelial functions by MVF and other methods such as flow-mediated brachial artery vasodilation and forearm plethysmography have been used for cardiovascular hazard identification of PM [27], whereas systemic inflammation and monocyte activation attachment to the endothelium is an important event in the atherosclerotic process [28]. As recently reported, active air filtration in the home had no effect on these outcomes, although the achieved reduction of PM_2.5_ in the bedroom was associated with an improvement in MVF [26]. For the present panel study, the outdoor air pollution exposure was assessed by urban background monitoring in terms of PM_10_, PM_2.5_, and PNC (size range 10–280 nm), which is highly dominated by UFP, whereas the indoor exposure assessment included week-based measurements of PM_2.5_, 24-h measurements of PNC (size range 10–300 nm) also highly dominated by UFP and presence of bio-aerosol components in settled dust on a 2-week basis. The goal of the study was to compare cardiovascular and respiratory effects associated with outdoor and indoor ultrafine, fine and microbiologically-derived PM among elderly subjects, using a panel design, limiting influence of individual confounders.

## 2. Materials and Methods

The study protocol was approved by the Committees on Health Research Ethics in the Capital Region of Denmark (H-4-2010-102), in accordance with the Declaration of Helsinki. All participants gave written informed consent prior to enrolment in the study.

### 2.1. Study Subjects and Design

We used repeated measurements of health outcomes and exposures in middle-aged participants, who participated in an air filtration study in their homes which were 27 apartments in Copenhagen, Denmark as described previously [26]. Briefly, 51 non-smoking volunteers (22 couples and seven singles) aged over 51 years from 29 apartments were recruited. One couple and a single person dropped out, leaving a study population of 48 subjects (21 couples and six singles) from 27 apartments. Of the subjects 22 were men and 26 were women. They were 67 ± 7 (mean ± SD) years old, had body mass index of 25 ± 3 kg/m^2^ and blood pressure and blood lipids in the normal range [26]. Eleven participants were taking vasoactive drugs (angiotensin-converting enzyme (ACE) inhibitors, calcium channel blockers, or β-adrenoreceptor blockers), 11 participants were taking statins and 12 participants were taking cyclooxygenase inhibitors (acetyl salicylic acid, ibuprofen or paracetamol). Two participants had been diagnosed with asthma and one participant had diabetes treated with metformin. The study lasted from November 2010 to May 2011 and involved 7 home visits over a 4-week period in each apartment. The visits were performed at day 1, 3, 8, 15, 17, 22 and 29 in one to three apartments per day, for measurements of the participant’s MVF and lung function and collection of blood samples in their homes. Air filtration units placed in the living room and bedroom were active from day 1 to 15 or from day 15 to 29 in random order as described previously [26]. Measurements of PM were made at indoor home sites of each participant and at an outdoor central monitoring station.

### 2.2. Exposure Assessment

Outdoor air pollution data were measured daily as part of the Danish Air Quality Monitoring Programme [29] at a Copenhagen urban background monitoring station within 5 km of all the homes in the study. The measurements included 24-h averages of PNC in the size range between 10 and 280 nm in mobility diameter (custom-built Differential Mobility Particle Sizer), PM_2.5_ and PM_10_ mass concentrations (SM200 instruments, OPSIS AB; Furulund, Sweden). The average concentration of the outdoor air pollutants during the 48 h prior to each set of health-related measurements was used as exposure estimate.

Indoor air pollution data were measured as previously described [26]. The level of indoor PM_2.5_ was measured in the bedroom and living room of each home on a weekly basis, *i.e*., with air sampling running from day 1 to 8, day 8 to 15, day 15 to 22 and day 22 to 29. BGI 400 pumps collected air samples continuously at a constant flow rate (4 L min^−1^ ± 10%) through cyclone sampling heads GK 2.05-KTL (BGI Incorporated, Waltham, MA, USA). Suspended matter in the PM_2.5_ range was separated on Zefluor W/PAD 37 mm filter membranes (Sigma-Aldrich, Brøndby, Denmark).

Indoor PNC were continuously monitored for 24 h in the living room of each home up to the health-related measurements on day 1, 15 and 29 with a Philips NanoTracer1000 (Philips Aerasense, Eindhoven, The Netherlands). This instrument has a time resolution of 16 s and detects PNC and average diameter of particles between 10 and 300 nm according to the manufacturer.

Indoor settled dust was also collected on a biweekly basis (from day 1 to 15 and day 15 to 29), by Electrostatic Dust Fall Collectors (EDC). The EDCs, consisting of a polypropylene folder with two electrostatic cloths (19 × 11 cm) (ZEEMAN, Alphen, The Netherlands), were placed in the living room on an open surface at ≥1 m above the floor level. The dust samples were analyzed for bacteria, endotoxin, fungi, serine protease and NAGase, expressed per EDC surface area per day, as previously described [30].

Due to prolonged sampling time required for collection of sufficient PM_2.5_ and dust material and a limited number of Nanotracer instruments, indoor exposure measurements could not be performed exactly prior to all the health-related measurements. Accordingly, the health-related and indoor PM measurements were matched as outlined in Table 1.

**Table 1:** Sampling periods used for exposure assessment in relation to 7 repeated health-related measurements.

Day of Biological Endpoint Sampling ^a^	1	3	8	15	17	22	29
Outdoor pollutants	48 h prior	48 h prior	48 h prior	48 h prior	48 h prior	48 h prior	48 h prior
Indoor PM_2.5_ living and bedroom		Day 1–8	Day 1–8	Day 8–15	Day 15–22	Day 15–22	Day 22–29
Indoor PNC	24 h prior			24 h prior			24 h prior
Indoor settled dust		Day 1–15	Day 1–15	Day 1–15	Day 15–29	Day 15–29	Day 15–29

Notes: ^a^ Including microvascular function (MVF), lung function, blood leukocyte counts, monocyte expression of adhesion molecules, C-reactive protein, Clara cell protein (CC16) and surfactant protein-D (SPD). PNC: particle number concentration; PM_2.5_: particulate matter with aerodynamic diameter less than 2.5 µm.

### 2.3. Measurement of Microvascular- and Lung Function

MVF was measured non-invasively via peripheral arterial tonometry using the portable EndoPAT 2000 (Itamar Medical Ltd, Cesaria, Israel), as previously described [26]. Vascular function changes in the digital pulse waveform signal detected by finger-mountable pneumatic sensors were elicited by creating a downstream hyperemic response after release of 5 min ischemia induced by inflation of a blood pressure cuff above systolic pressure, whereas the digital signal of the contralateral index finger served as reference. The response to reactive hyperemia was calculated automatically through a computer algorithm and a reactive hyperemia index was created by the ratio of the post and pre occlusion values of the pulse waveform signals.

The lung function was measured by spirometry in accordance with the American Thoracic Society/European Respiratory Society standard guidelines [31] using the EasyOne Plus spirometer (ndd Medical Technologies; Zurich, Switzerland) as previously described [26]. The spirometric measures of forced expiratory volume in first second (FEV_1_) and forced vital capacity (FVC) were collected after MVF measurements. The data were digitally stored and the largest FVC and FEV_1_ from at least three acceptable trials were used; the ratio of FEV_1_ to FVC was calculated.

### 2.4. Measurement of Biomarkers

On the day of the home visits, peripheral venous blood samples were collected in CPT^TM^ tubes with sodium heparin (BD Vacutainer^®^ CPT™, Becton Dickinson A/S, Brøndby, Denmark) for peripheral blood mononuclear cells (PBMC) isolation and in EDTA tubes for hematological analyses as previously described [26]. Measurements of hemoglobin, and leukocyte counts and their differential profile (lymphocytes, monocytes, granulocytes) were performed by an automatic hematological analyzer, Chempaq (Chempaq XBC, Copenhagen, Denmark)

The concentrations of CC16 and SPD in plasma were analyzed by ELISA (Human Clara Cell Protein ELISA kit from BioVendor Laboratory Medicine, Inc., Brno, Czech Republic) at the Department of Occupational and Environmental Medicine, Sahlgrenska University Hospital and Sahlgrenska Academy, University of Gothenburg.

Plasma CRP, total cholesterol, high-density lipoprotein (HDL), low-density lipoprotein (LDL) and triglycerides were analyzed at the Department of Clinical Biochemistry, Copenhagen University Hospital.

Direct immunofluorescence of PBMC was performed on a BD Accuri™ C6 flow cytometer with BD Accuri CFlow^®^Plus software (BD Bioscience, Brøndby, Denmark) as previously described [26]. Briefly, specific surface staining of the activation status of monocytes was performed with fluorescein isothiocyanate (FITC)-conjugated anti-CD49d (ITGA4), +Allophycocyanin (APC)-conjugated anti-CD11b (Mac1α) and FITC-conjugated anti-CD31 (PECAM-1) + Phycoerythrin (PE)-conjugated anti-CD62L (L-selectin) mouse monoclonal antibodies (BD Bioscience). PBMC were placed in round-bottom 96-well plates (approximately 10^5^ cells per well), stained for 30 min at 4 °C, washed twice with stain buffer with centrifugation at 250 g for 5 min, resuspended in 100 µL stain buffer and analyzed immediately. Monocytes were selectively gated based on their characteristic forward scatter and side scatter properties. The expression of CD11b, CD31, CD62L and CD49d on monocytes was quantified as percentage of positive cells from each sample.

### 2.5. Statistical Analysis

Pearson correlation coefficients were calculated between the simultaneously determined indoor and outdoor pollutant levels. Linear mixed models (*xtmixed* procedure) were used to estimate the association between log-transformed health outcomes and indoor and outdoor exposure variables, accounting for correlation between repeated measurements within individuals and for correlation between individuals living at the same address. Separate models were fitted for each outcome, adjusted for age, gender, BMI, active or no indoor air filtration and day of measurement. For the outdoor exposure variables, models were further adjusted for potential temporal confounders such as outdoor temperature and season of measurement. We also tested whether indoor air filtration modified associations between exposures and outcomes by introducing an interaction term in the analyses. Additionally, we assessed whether the associations between the exposure and health outcomes were affected by intake of any drug by further adjustment for this in the analyses and by analyses stratified by drug intake with adjustment for age, gender, BMI, indoor filtration and day of measurements.

Results were expressed as percentage change with 95% confidence intervals of an outcome per increase in a pollutant’s interquartile range (IQR) concentration. We used the IQRs in the analysis of the indoor and the outdoor data pollutants to allow direct comparison of effect estimates. A value of *p* ≤ 0.05 was considered statistically significant. Analyses were performed using STATA software (version 12.0, StataCorp LP, College Station, TX, USA).

## 3. Results

### 3.1. Exposure Characterization

Table 2 and Table 3 outline the results of the 4-week indoor air monitoring of the 27 apartments for PNC, PM_2.5_ and the level of endotoxin, fungi, bacteria, serine protease and NAGase in settled dust. The indoor PNC, PM_2.5_ and level of bacteria, fungi and serine protease in settled dust were reduced during active as compared with inactive air filtration, whereas there was no significant effect on endotoxin or NAGase (Table 2). The ambient air PNC, PM_2.5_ and PM_10_ concentrations, monitored at an urban background station are summarized in Table 3. There were no significant correlations between simultaneously measured indoor and outdoor PNC (r = −0.11), whereas indoor levels of PM_2.5_ in the bedroom and outdoor PM_2.5_ showed weak, but significant, positive correlation (r = 0.20). The levels of bacteria, endotoxin and fungi in indoor settled dust were weakly but significantly correlated with indoor levels of PM_2.5_ in the bedroom and living room (r = 0.18–0.33). There were also significant correlations between levels of bacteria, endotoxin and serine protease in indoor settled dust. Outdoor levels of PNC and especially PM_2.5_ and PM_10_ were, as expected, more strongly and significantly correlated (Table 3).

### 3.2. Physiological Functions and Biomarkers

The associations between the health outcomes and the indoor and outdoor air pollutants estimated as percent change per IQR are presented in Table 4. We found that the outdoor PNC was significantly inversely associated with MVF, whereas a similar association with indoor PNC was not statistically significant. The levels of outdoor PM_2.5_ were significantly inversely associated with counts of granulocytes. The level of bacteria in indoor settled dust was significantly positively associated with MVF, the levels of fungi and serine protease in indoor settled dust were associated with a significant decrease in expression of adhesion marker CD31 on monocytes, whereas the PM_2.5_ concentration in bedroom and the levels of endotoxin and bacteria in settled dust were associated with a significant increase in CD11b expression on monocytes. Plasma levels of CC16 and SPD showed significant positive associations with the levels of NAGase and endotoxin in settled dust, respectively. The lung function was inversely associated with PNC both indoors and outdoors, although none reached statistical significance.

**Table 2:** Exposure levels of particles and settled dust bio-aerosols in the living or bedroom of the participants during sham and active indoor air filtration.

Indoor Air Pollutants	Filtration		Mann-Whitney Test
	Sham	Active	*p*-Values
PNC (#/cm^3^) in LR	7669 (3435, 45,866)	5618 (1241, 56,654)	0.08
PM_2.5__total mass ^a^ (µg/m^3^) in LR	8.0 (3.4, 20.7)	4.3 (0.2, 12.2)	0.00
PM_2.5__total mass ^a^ (µg/m^3^) in BR	7.6 (1.4, 19.1)	3.7 (0, 14)	0.00
Bacteria (CFU/m^2^/day) in LR	2529 (458, 10,606)	2098 (159.5, 6826)	0.02
Endotoxin (EU/m^2^/day) in LR	111 (33.3, 502)	126 (35.9, 374)	0.85
Fungi (CFU/m^2^/day) in LR	1743 (398.7, 5366)	1094 (205, 2939)	0.00
Serine protease (µg/m^2^/day) in LR	51.3 (12.0, 93.3)	56.4 (20.1, 96.4)	0.04
NAGase (pmol 4-MU/m^2^/day) in LR	3453 (388, 10,049)	3841 (388, 12144)	0.15

Notes: Values are median (5th, 95th percentile). ^a^ PNC (particle number concentration) and PM_2.5_ (particulate matter less than 2.5 µm) total mass data have recently been reported elsewhere [26,32]. BR: bedroom, LR: living room.

The significant associations were robust to further adjustment for intake of any drug (Table 5). In analysis stratified for intake of any drug, the associations had with one exception (CC16 and indoor levels of NAGase) the same direction and widely overlapping confidence intervals, although most were statistically significant in only one group and two associations were not statistically significant within either group. Of particular note, the inverse association between MVF and outdoor PNC levels was of quite similar magnitude in subjects taking and not taking any drug, but not statistically significant in any of the groups. None of the interaction terms related to drug intake were statistically significant.

## 4. Discussion

Our study shows a negative association between MVF and outdoor/urban background PNC, but not with outdoor levels of PM_10_ or PM_2.5_, which was inversely associated with granulocyte counts. The exposure to indoor PM_2.5_ showed positive association with monocyte activation marker CD11b, whereas bio-aerosols were positively associated with monocyte activation markers, MVF and biomarkers of epithelial integrity in the lower airways.

**Table 3:** Median (5th, 95th percentile) of all measurements and correlation coefficients (*p*-values) of simultaneously measured indoor and outdoor air pollutants.

Exposure Variables		Indoor Exposure and Sampling Averaging Time								Outdoor		
		PNC 24 h (10^3^/cm^3^)	PM_2.5_LR 7 days (µg/m^3^)	PM_2.5_BR 7 days (µg/m^3^)	Bacteria 14 days (CFU/m^2^/day)	Endotoxin 14 days (EU/m^2^/day)	Fungi 14 days (CFU/m^2^/day)	Serine Protease 14 days (µg/m^2^/day)	NAGase 14 days (pmol 4-MU/m^2^/day)	PNC 48h prior (10^3^/cm^3^)	PM_2.5_ 48h prior (µg/m^3^)	PM_10_ 48h prior (µg/m^3^)
	Median (5th, 95th percentile)	7.1 (1.7, 94.7)	6.3 (1.8, 17)	6.3 (0, 18)	2529 (205, 10,606)	115 (33.4, 477)	1196 (205, 5366)	54.7 (16, 94)	3841 (388, 12,144)	5.4 (3.0, 10.4)	15 (5.2, 42.3)	22 (7.5, 47)
Indoor	PNC	1.0000										
	PM_2.5_ LR		1.0000									
	PM_2.5_ BR		0.65 * (0.00)	1.0000								
	Bacteria		0.18 * (0.03)	0.27 * (0.00)	1.0000							
	Endotoxin		0.33 * (0.00)	0.32 * (0.00)	0.58 * (0.00)	1.0000						
	Fungi		0.20 * (0.01)	0.21 * (0.01)	−0.07 (0.38)	−0.08 (0.36)	1.0000					
	Serine protease		0.05 (0.60)	0.16 (0.05)	0.29 * (0.00)	0.19* (0.02)	−0.11 (0.19)	1.0000				
	NAGase		−0.11 (0.18)	0.03 (0.70)	0.13 (0.12)	−0.08 (0.35)	−0.06 (0.47)	−0.09 (0.31)	1.0000			
Outdoor	PNC	−0.11 (0.33)	0.00 (0.98)	0.03 (0.69)	−0.19 * (0.02)	−0.13 (0.11)	−0.04 (0.65)	−0.05 (0.58)	0.13 (0.11)	1.0000		
	PM_2.5_	−0.12 (0.29)	0.13 (0.14)	0.20 * (0.02)	−0.12 (0.14)	−0.02 (0.83)	0.03 (0.73)	−0.06 (0.45)	0.00 (0.97)	0.45 * (0.00)	1.0000	
	PM_10_	−0.08 (0.47)	0.05 (0.56)	0.15(0.08)	−0.15 (0.07)	−0.07 (0.39)	0.06 (0.51)	−0.12 (0.14)	0.00 (0.96)	0.46 * (0.00)	0.90 * (0.00)	1.0000

Notes: PNC: particle number concentration; PM_2.5_: particulate matter less than 2.5 µm; LR in living room; BR: in bedroom; EU: endotoxin units (12 EU = 1 ng); CFU: colony forming units; 4-MU: 4-methylumbelliferone. * *p* < 0.05.

**Table 4:** Percent changes (95% confidence interval) in outcome levels associated with one interquartile range increase (IQR) in indoor and outdoor exposures, estimated by mixed-effects models with the natural logarithm of the outcomes, accounting for correlation between repeated measurements within subjects and with subjects nested in residence; all models were adjusted for age, gender, BMI, indoor filtration and days of measurements. Associations with outdoor pollutants were further adjusted for outdoor temperature and season.

Outcome Variables Median (95% Confidence Interval)	Indoor Exposure Characteristics (IQR)								Outdoor Exposure Characteristics (IQR)		
	PNC (13.6 10^3^/cm^3^)	PM_2.5_ LR (5.7 µg/m^3^)	PM_2.5_ BR (6.1 µg/m^3^)	Bacteria (3383 CFU/m^2^/day)	Endotoxin (111 EU/m^2^/day)	Fungi (1934 CFU/m^2^/day)	Serine protease (27.5µg/m^2^/day)	NAGase (4826 pmol4−MU/m^2^/day)	PNC (3.010^3^/cm^3^)	PM_2.5_ (11.9 µg/m^3^)	PM_10_ (14.0 µg/m^3^)
**MVF**	−2.1	−1.1	1.2	5.6 *	1.7	−2.8	2.6	−1.1	−3.4 *	−1.2	−0.5
1.73 (1.25, 2.79)	(−4.9, 0.8)	(−4.7, 2.5)	(−2.2, 4.9)	**(2.6, 8.7)**	(−0.3, 3.8)	(−6.7, 1.2)	(−2.4, 7.8)	(−4.0, 1.8)	**(−6.6, −0.05)**	(−4.1, 1.8)	(−3.9, 2.9)
**C−reactive protein**	−4.2	−1.3	−2.3	−0.7	3.0	4.2	4.1	0.2	3.4	−2.8	−4.3
0.9 (0.2, 4.7; mg/L)	(−13.7, 6.2)	(−10.6, 8.9)	(−11.3, 7.5)	(−9.4, 8.9)	(−2.8, 9.2)	(−7.8, 17.9)	(−14.8, 27.4)	(−8.4, 9.8)	(−6.2, 13.9)	(−10.6, 5.7)	(−13.2, 5.5)
**Leukocytes** 5.7 (3.9, 8.3; 10^9^ cells/L)	−0.6 (−3.3, 2.2)	−1.7 (−4.4, 0.9)	−0.6 (−3.2, 2.0)	0.8 (−1.6, 3.2)	0.5 (−1.0, 2.1)	2.6 (−0.7, 5.9)	−3.5 (−8.2, 1.5)	0.5 (−1.9, 2.9)	0.8 (−1.8, 3.4)	−2.1 (−4.2, 0.1)	−1.9 (−4.4, 0.8)
**Lymphocytes** 2.0 (0.9, 3.3; 10^9^ cells/L)	−1.7 (−5.3, 2.1)	−2.9 (−6.1, 0.5)	0.6 (−2.7, 4.0)	−0.9 (−3.9, 2.1)	0.9 (−1.1, 2.9)	0.2 (−3.9, 4.5)	−4.0 (−10.4, 2.7)	0.6 (−2.5, 3.8)	1.7 (−1.6, 5.0)	2.3 (−0.5, 5.3)	2.1 (−1.2, 5.6)
**Monocytes** 0.6 (0.4, 0.9; 10^9^ cells/L)	−1.4 (−4.3, 1.5)	−1.0 (−3.7, 1.8)	0.8 (−1.8, 3.6)	1.4 (−1.1, 3.9)	1.2 (−0.4, 2.9)	1.2 (−2.2, 4.5)	−1.6 (−6.8, 3.7)	1.1 (−1.4, 3.7)	2.0 (−0.6, 4.8)	−0.2 (−2.5, 2.1)	−0.2 (−2.9, 2.5)
**Granulocytes** 2.9 (1.8, 5.4; 10^9^ cells/L)	0.2 (−3.4, 3.9)	−0.6 (−4.5, 3.5)	−1.3 (−5.1, 2.7)	2.2 (−1.3, 5.8)	0.05 (−2.2, 2.4)	3.2 (−1.6, 8.3)	−2.1 (−8.8, 5.1)	0.5 (−3.0, 4.2)	0.4 (−3.4, 4.4)	−4.1 * (−7.3, −0.9)	−3.3 (−6.9, 0.5)
**CD31** 92.9 (82.1, 97.9; %)	−0.01 (−1.1, 1.1)	0.1 (−1.6, 1.8)	−1.3 (−2.9, 0.3)	0.4 (−0.9, 1.7)	0.6 (−0.3, 1.5)	−3.1 * (−4.8, −1.3)	−2.3 * (−3.9, −0.7)	−0.2 (−1.5, 1.1)	−0.2 (−1.9, 1.5)	0.8 (−0.7, 2.3)	0.05 (−1.6, 1.7)
**CD62** 62.4 (41.9, 79.1; %)	0.2 (−2.4, 3.0)	2.5 (−0.6, 5.8)	0.2 (−2.9, 3.4)	1.5 (−1.2, 4.3)	1.6 (−0.2, 3.4)	−2.9 (−6.5, 0.8)	−1.6 (−6.5, 3.4)	−2.7 (−5.3, 0.003)	−0.7 (−3.7, 2.4)	2.7 (−0.03, 5.4)	1.3 (−1.8, 4.5)
**CD11b** 38.3 (6.7, 70.0; %)	1.6 (−6.2, 10.1)	1.3 (−6.9, 10.1)	9.1 * (0.6, 18.3)	8.2 * (0.4, 16.6)	8.1 * (3.0, 13.4)	−3.9 (−13.1, 6.3)	5.9 (−10.3, 25.1)	2.6 (−4.9, 10.7)	4.3 (−3.7, 13.0)	3.1 (−3.7, 10.4)	0.4 (−7.2, 8.6)
**CD49** 71.7 (32.6, 95.6; %)	−0.8 (−4.2, 2.7)	−1.0 (−5.5, 3.8)	3.1 (−1.4, 7.9)	1.8 (−2.3, 6.1)	1.3 (−1.4, 4.0)	4.9 (−0.7, 10.9)	2.0 (−5.7, 10.4)	0.4 (−3.7, 4.5)	1.0 (−3.4, 5.7)	1.0 (−2.8, 5.1)	0.3 (−4.2, 5.0)
**FEV1/FVC** 0.73 (0.41, 1.24)	−2.9 (−5.8, 0.1)	0.9 (−3.7, 5.8)	−2.0 (−6.2, 2.5)	1.5 (−2.2, 5.3)	−0.2 (−2.7, 2.4)	−2.4 (−7.4, 2.9)	−1.2 (−7.2, 5.2)	−1.0 (−4.7, 2.8)	−4.0 (−8.1, 0.5)	−1.9 (−5.5, 1.8)	−1.7 (−5.9, 2.7)
**CC16** 4.0 (2.0, 9.4; ng/mL)	−0.7 (−5.1, 3.9)	−0.6 (−5.5, 4.5)	−2.4 (−7.2, 2.5)	−1.3 (−5.8, 3.3)	−1.5 (−4.5, 1.5)	3.8 (−2.5, 10.4)	4.7 (−5.7, 16.2)	5.2 * (0.3, 10.4)	2.1 (−2.8, 7.3)	0.6 (−3.6, 5.0)	0.2 (−4.7, 5.3)
**SPD** 98.7 (46.2, 302.8; ng/mL)	−0.2 (−3.5, 3.2)	−0.3 (−3.3, 2.8)	−1.2 (−4.2, 1.7)	−0.9 (−3.7, 1.8)	−2.2 * (−4.0, −0.5)	−1.3 (−4.9, 2.5)	−4.5 (−11.2, 2.7)	2.7 (−0.2, 5.7)	0.5 (−2.3, 3.5)	0.2 (−2.3, 2.8)	1.1 (−1.8, 4.2)

Notes: Abbreviations: MVF: microvascular function; CD: cluster of differentiation; FEV1: forced expiratory volume in first second; FVC: forced vital capacity; PNC: particle number concentration; PM_2.5_: particulate matter less than 2.5 µm; _LR in living room; _BR: in bedroom; CFU: colony forming units; EU: endotoxin units (12 EU = 1 ng); 4-MU: 4-methylumbelliferone; PM_10_: particulate matter less than 10 µm. SPD: surface protein D. * *p* < 0.05.

**Table 5:** Percent changes (95% confidence interval) in outcome levels associated with one interquartile range increase in indoor and outdoor exposures, estimated by mixed-effects models with the natural logarithm of the outcomes, accounting for correlation between repeated measurements within subjects and with subjects nested in residence; all models were adjusted for age, gender, BMI, indoor filtration and days of measurements. Only associations statistically significant as outlined in Table 4 are included and here presented without and with further adjustment for intake of any drug and stratified into groups of subjects not taking or taking any drug.

Outcome Variable	Exposure Variable	Percent Change without Adjustment for Drug Intake	Percent Change with Adjustment for Drug Intake	Percent Change in 25 Subjects without Any Drug Intake	Percent Change in 23 Subjects with Any Drug Intake	*p*-Value for Interaction with Drug Intake
MVF	Indoor Bacteria	5.6 * (2.6, 8.7)	5.6 * (2.6, 8,7)	9.1 * (1.4, 17,4)	4.9 * (1.8, 8,1)	0.22
MVF	Outdoor PNC	−3.4 * (−6.6, −0.05)	−3.4 * (−6.6, −0.1)	−2.3 (−7.1, 2.8)	−3.9 (−8.2, 0.5)	0.82
Granulocytes	Outdoor PM_2.5_	−4.1 * (−7.3, −0.9)	−4.1 * (−7.3, −0.9)	−5.4 * (−10.1, −0.5)	−2.9 (−7.0, 1.4)	0.27
CD31	Indoor Fungi	−3.1 * (−4.8, −1.3)	−2.9 * (−4.6, −1.2)	−1.0 (−3.1, 1.1)	−3.7 * (−6.1, −1.3)	0.17
CD31	Indoor Serine protease	−2.3 * (−3.9, −0.7)	−2.4 * (−3.9, −0.9)	−1.5 (−3.0, 0.1)	−2.4 (−4.8, 0.1)	0.57
CD11b	Indoor PM_2.5_ BR	9.1 * (0.6, 18.3)	**9.3 (0.8, 18.5)**	2.5 (−9.0, 15.4)	16.4 * (4.4, 29.7)	0.21
CD11b	Indoor Bacteria	8.2 * (0.4, 16.6)	8.1 * (0.4, 16.5)	5.2 (−10.9, 24.4)	9.2 * (0.1, 19.0)	0.97
CD11b	Indoor Endotoxin	8.1 * (3.0, 13.4)	8.1 * (3.1, 13.4)	5.1 (−1.2, 11.9)	9.9 * (2.5, 17.9)	0.35
CC16	Indoor Nagase	5.2 * (0.3, 10.4)	5.2 * (0.3, 10.4)	−1.5 (−8.0, 5.3)	6.0 (−0.9, 13.5)	0.27
SPD	Indoor Endotoxin	−2.2 * (−4.0, −0.5)	−2.2 * (−4.0, −0.5)	−1.0 (−3.5, 1.6)	−3.2 * (5.6, −0.9)	0.33

Notes: Abbreviations: MVF: microvascular function; CD: cluster of differentiation; FEV1: forced expiratory volume in first second; FVC: forced vital capacity; PNC: particle number concentration; PM_2.5_: particulate matter less than 2.5 µm; BR: in bedroom; PM_10_: particulate matter less than 10 µm. * *p* < 0.05.

The negative association between the outdoor PNC levels and MVF is consistent with results from a cross sectional study, which we have conducted a year after the present data collection, using PNC levels at the same urban background monitoring station. Among 80 healthy middle aged subjects living in the same Copenhagen area, an IQR change in the urban background PNC level monitored for 48 h up to MVF measurements was associated with an 8% decrease in MVF [24]. Like in the present study that association did not appear to be affected by intake of drugs [24], although only the subjects not taking any drugs appeared to have improved MVF in relation to the actual decrease in bedroom PM_2.5_ achieved by air filtration in the intervention related analysis of the present material [26]. Especially vasoactive drugs like ACE-inhibitors, calcium antagonists and beta-blockers can affect MVF and thus possibly blur associations with PM exposure. Moreover, in a second cross sectional study conducted yet a year later, among 81 middle-aged subjects living in the western part of Copenhagen, we used personally monitored PNC for 48 h for exposure assessment in 59 subjects [25]. Their integrated exposure when away from home during the 48-h monitoring period was inversely associated with MVF. These two cross-sectional studies were susceptible to individual confounders which is not the same problem in the present panel study with repeated measures design. Accordingly, we have three independent observations in 3 different populations of middle-aged and elderly citizens of Copenhagen showing that MVF is inversely associated with outdoor PNC levels or personal PNC levels away from home. This is consistent with observations that short-term exposure to diesel combustion-related particles adversely affects endothelial function [33,34]. Similarly, high levels of ambient PM_2.5_ in the preceding two days was associated with low MVF in a 3-h controlled exposure study [35]. In contrast, a previous study of young healthy adults found no effect on MVF following a 24-h exposure period to air from above a street with heavy traffic in Copenhagen [36].

We found an inverse, but not significant, association between MVF and the indoor air PNC monitored for 24 h before the measurement, whereas there were inconsistent associations with PM mass in living room and bedroom, which however, could not be monitored in the 48-h intervals before measurements and short-term associations might have been missed. Indeed, two short-term intervention studies with filtration of indoor air resulting in 60% to 70% decreases in PNC and/or PM_2.5_ for 2–7 days, resulted in increased MVF in young adults and elderly inhabitants, respectively [37,38]. Similarly, our intervention study with filtration of indoor air for 2 weeks which the present panel study is based on, showed a significant improvement in MVF only in relation to the actual decrease in PM_2.5_ in the bedroom within 2 days [26].

The significant positive association between the level of bacteria in settled dust and MVF was unexpected, although we have not found any publications relating inhalation exposure to microbial compounds and vascular function in human subjects. However, very high level exposure in terms of intravenous administration of endotoxin in single doses decreased endothelial function although tolerance developed with repeated dosing in human experiments [39]. Nevertheless, the level of bacteria in settled dust in these homes was in the lower end of the range found in Central Europe and most are Gram negative without endotoxin production [30,40]. Interestingly, only the levels of bacteria, fungi and serine protease were affected by the indoor air filtration, whereas their derived components endotoxin, and NAGase showed no change.

Multiple studies have shown that high leukocyte counts and their subtypes can be biomarkers of vascular inflammation and predictors of cardiovascular disease risk [41]. We found significant inverse associations between outdoor levels of PM_2.5_ and granulocytes, which is consistent with a decrease in circulating leukocytes after exposure to ambient air PM [42] or concentrated ambient air particles [43]. In our earlier cross-sectional study we also found a non-significant inverse association between outdoor PM_2.5_ for 48 h prior to blood sampling and total leukocytes (2.3% lower per IQR; 95%CI: −1% to 5.5%) and neutrophils (3.4% lower per IQR; 95%CI: −0.2% to 6.9%). In contrast, no effects on leukocytes or granulocytes were found after exposures to concentrated ambient air [44], diesel exhaust [45,46,47,48] or to concentrated ambient UFP [48]. No consistent association between exposure to ambient PM and lymphocytes and monocytes were reported in a recent study on in-traffic exposure in healthy adults [49], while other studies have reported increased levels of circulating leukocytes in blood samples from subjects in the general population and patients with chronic pulmonary diseases after short-term increases in ambient air PM levels [50,51].

We also investigated the expression of surface adhesion molecules on circulating monocytes by flow cytometry, because monocyte activation with adherence to the endothelium is an important event in the atherosclerotic process [28]. The expression of adhesion markers CD31 on monocytes was significantly inversely associated with indoor fungi and serine protease levels, while the expression of adhesion markers CD11b on monocytes showed a significantly positive association with PM_2.5_ in the bedroom, indoor endotoxin and bacteria levels. However, in our earlier cross-sectional study similar associations with CD11b were inverse, the association with indoor endotoxin even statistically significant and only between CD31 and fungi did we find similar association (0.7% lower per IQR; 95%CI: −0.7% to 2.1%) [24]. This suggests that the present associations could be spurious or that systemic inflammation responses can be affected by the levels of indoor PM_2.5_ and some of bio-aerosols. Earlier studies on exposure to PM or air pollution have shown effects on several of these molecules. For instance, particle exposure to carbon UFP in healthy subjects was associated with reduced expression of adhesion molecules CD11b/CD18 on monocytes and CD11b/CD18 and CD49d on granulocytes [52]. Furthermore, an animal experimental study showed that exposure to PM air pollution was associated with accelerated recruitment of monocytes into atherosclerotic plaques, possibly by upregulating the expression of the adhesion molecules involved in monocyte recruitment [53]. The exposed animals in that study also showed decreased circulating monocytes expressing high levels of CD31 (PECAM-1) and decreased numbers of monocytes with CD49d (very late antigen-4 α-chain) in atherosclerotic plaques [53]. Moreover, one week’s exposure to biomass smoke in a reconstructed Viking house was associated with increased expression of CD31 on monocytes [54], whereas chronic biomass smoke exposure was associated with increased surface expression of CD11b/CD18 in circulating polymorphonuclear leukocytes and monocytes in Indian women [55]. On the other hand, 3-h inhalation exposure to high concentrations of wood smoke particles had no apparent effects on surface marker molecules CD54 (ICAM-1), CD11a (ITGAL) and CD62L (L-selectin) at 6 or 20 h after cessation of the exposure [56].

A significant negative association between indoor PNC and lung function (2% per IQR increase) was found in the 78 participants of our previous cross-sectional study [24]. In the present study population, we also found that lung function was inversely associated with indoor PNC (2.9% decrease in FEV_1_/FVC per IQR increase in PNC) as well as PM_2.5_ in the bedroom and outdoor PNC (4% decrease per IQR increase) and PM mass albeit none were significant. In wintertime in Denmark the variation in indoor PNC is dominated by candle burning [16,24], which is also emitting nitrogen dioxide that could contribute to adverse effects on lung function. Adverse effects of emission from candle burning on lung function has been further supported by a recent controlled exposure study where FEV_1_ decrements were associated with particle mass and surface area from burning candles [57]. Our most recent cross-sectional study showed a weak inverse association between lung function and personally monitored PNC but no association with stationary monitoring of indoor PNC in the home in the spring period, when candle burning is much less than in winter time [25]. There appear to be no other studies relating indoor PNC to lung function, whereas studies relating lung function to indoor PM_2.5_ mainly show no associations in healthy individuals [58,59,60].

The plasma levels of CC16 and SPD were used as biomarkers for assessing adverse effects on the cellular integrity or the permeability of the lung epithelium, where short-term exposure to ambient PM_10_, wood smoke, pure endotoxin and high occupational exposure to bacterial components have mainly been associated with elevated serum levels of especially CC16, whereas long-term exposure has shown no or even inverse associations [61,62,63,64,65,66]. Short-term exposure to traffic-polluted air has shown no apparent effect on CC16 [49,67]. We only found significant associations between the level of CC16 and NAGase and the level of SPD and the indoor level of endotoxin, where the latter was inversely associated, whereas the former association had opposite direction in subjects taking and not taking any drugs. Accordingly, the significant associations between bio-aerosol components and pneumoproteins in our material may well be spurious and the temporal variability in the level of all the exposures might have been too low to provide detectable associations with the pneumoproteins.

We used a robust panel study design, with 7-repeated measurements on the same individual, in a real-life setting. This facilitated assessment of the short-term association between variation in indoor and outdoor air pollution and health-related outcomes over time, within individuals. The limitations of this study include the small sample size and heterogeneous study population aged 51 to 81 years with some participants taking vasoactive and/or cyclooxygenase inhibitor drugs. Another limitation is the timing of the measurements used for assessment of indoor exposures as it was not possible to have specific monitoring preceding each of the seven sets of outcome measurement per participant and some of the temporal variability was lost. Moreover, we had only quantitative assessment of whole groups of microorganisms, where different species might have very different biological activities. We did not have information on behavior affecting exposure and sufficiently detailed time-activity pattern for assigning the participants to specific microenvironments to improve assessment of source specific exposure both in their home and out of their home for which we could only use adjustment for time spend at home. Furthermore, we used outdoor exposure data collected at a central monitoring site, which is likely to cause measurement error for exposure to PNC that show substantial spatial variation, whereas PM_2.5_ and PM_10_ are more uniformly distributed over Copenhagen [68]. Accordingly, misclassification of exposure is likely to be substantial but most likely to be random and would thus rather tend to weaken associations. Furthermore, we tested a large number of associations between a series of outcomes (13) and a number of exposures (11), which would be expected to give rise to around 7 statistically significant associations at the 5% level by chance. However, we consider our study exploratory and have not adjusted levels of significance, but rather put more weight on associations related to MVF and lung function consistent with findings in our other studies with similar exposure assessment in other populations.

## 5. Conclusions

The study suggests that PNC in the outdoor environment is associated with decreased MVF, while the exposure to outdoor PM mass and PM and bio-aerosols in the indoor environment may be associated with some markers of inflammation and disruption of lung cell integrity. However, multiple comparisons were made and the results should be interpreted with caution. The decrease in MVF associated with outdoor PNC is consistent with our observations relating MVF to UFP outside the home in other Copenhagen populations. Although the observed changes associated with the air pollutants were small and the cardiovascular and respiratory functions stayed within the normal range, such changes could be of clinical importance among more susceptible individuals.

## Abbreviations

BMI: Body mass index
CC16: Clara cell pneumoprotein 16
CD: Cluster of differentiation
CRP: C-reactive protein
EDC: Electrostatic Dust Fall Collectors
FEV_1_: Forced expiratory volume in 1 second
FVC: Forced vital capacity
HEPA: High-efficiency particulate arrestance
IQR: Interquartile range
MVF: Microvascular function
NAGase: N-Acetyl-β-d-glucosaminidase
PBMC: Peripheral blood mononuclear cells
PM_2.5_: Particulate matter with aerodynamic diameter less than 2.5 μm
PNC: Particle number concentration
SPD: Surfactant protein D
UFP: Ultrafine particles
